# Chronic Treatment with Ivabradine Does Not Affect Cardiovascular Autonomic Control in Rats

**DOI:** 10.3389/fphys.2016.00305

**Published:** 2016-07-26

**Authors:** Fernanda C. Silva, Franciny A. Paiva, Flávia C. Müller-Ribeiro, Henrique M. A. Caldeira, Marco A. P. Fontes, Rodrigo C. A. de Menezes, Karina R. Casali, Gláucia H. Fortes, Eleonora Tobaldini, Monica Solbiati, Nicola Montano, Valdo J. Dias Da Silva, Deoclécio A. Chianca

**Affiliations:** ^1^Laboratory of Cardiovascular Physiology, Department of Biological Sciences, Institute of Exact and Biological Sciences, Federal University of Ouro PretoOuro Preto, Brazil; ^2^Graduate Program in Biological Sciences – CBIOL/NUPEB, Federal University of Ouro PretoOuro Preto, Brazil; ^3^Laboratory of Hypertension, Institute of Biological Sciences, Department of Physiology and Biophysics, Federal University of Minas GeraisBelo Horizonte, Brazil; ^4^Laboratory of Biomedical Engineering, Institute of Science and Technology, Federal University of São PauloSão José dos Campos, Brazil; ^5^Department of Physiology, University of UberabaUberaba, Brazil; ^6^Department of Clinical Sciences and Community Health, IRCCS Ca' Granda Foundation, Ospedale Maggiore Policlinico, University of MilanMilan, Italy; ^7^Department of Physiology, Institute of Biological and Natural Sciences, Federal University of Triângulo MineiroUberaba, Brazil

**Keywords:** ivabradine, HCN channels, renal sympathetic nerve activity, cardiovascular reflexes, tonic control, vagal activity, sympathetic activity

## Abstract

A low resting heart rate (HR) would be of great benefit in cardiovascular diseases. Ivabradine—a novel selective inhibitor of hyperpolarization-activated cyclic nucleotide gated (HCN) channels- has emerged as a promising HR lowering drug. Its effects on the autonomic HR control are little known. This study assessed the effects of chronic treatment with ivabradine on the modulatory, reflex and tonic cardiovascular autonomic control and on the renal sympathetic nerve activity (RSNA). Male Wistar rats were divided in 2 groups, receiving intraperitoneal injections of vehicle (VEH) or ivabradine (IVA) during 7 or 8 consecutive days. Rats were submitted to vessels cannulation to perform arterial blood pressure (AP) and HR recordings in freely moving rats. Time series of resting pulse interval and systolic AP were used to measure cardiovascular variability parameters. We also assessed the baroreflex, chemoreflex and the Bezold-Jarish reflex sensitivities. To better evaluate the effects of ivabradine on the autonomic control of the heart, we performed sympathetic and vagal autonomic blockade. As expected, ivabradine-treated rats showed a lower resting (VEH: 362 ± 16 bpm vs. IVA: 260 ± 14 bpm, *p* = 0.0005) and intrinsic HR (VEH: 369 ± 9 bpm vs. IVA: 326 ± 11 bpm, *p* = 0.0146). However, the chronic treatment with ivabradine did not change normalized HR spectral parameters LF (nu) (VEH: 24.2 ± 4.6 vs. IVA: 29.8 ± 6.4; *p* > 0.05); HF (nu) (VEH: 75.1 ± 3.7 vs. IVA: 69.2 ± 5.8; *p* > 0.05), any cardiovascular reflexes, neither the tonic autonomic control of the HR (tonic sympathovagal index; VEH: 0.91± 0.02 vs. IVA: 0.88 ± 0.03, *p* = 0.3494). We performed the AP, HR and RSNA recordings in urethane-anesthetized rats. The chronic treatment with ivabradine reduced the resting HR (VEH: 364 ± 12 bpm vs. IVA: 207 ± 11 bpm, *p* < 0.0001), without affecting RSNA (VEH: 117 ± 16 vs. IVA: 120 ± 9 spikes/s, *p* = 0.9100) and mean arterial pressure (VEH: 70 ± 4 vs. IVA: 77 ± 6 mmHg, *p* = 0.3293). Our results suggest that, in health rats, the long-term treatment with ivabradine directly reduces the HR without changing the RSNA modulation and the reflex and tonic autonomic control of the heart.

## Introduction

There is a clear association between increased resting heart rate (rHR) and mortality rate, especially in patients suffering from cardiovascular disease (Fox et al., 2008; Verrier and Tan, 2009). It is noteworthy that this association is true not only for very high values of rHR, since rHR upper than 83 bpm has been associated with an increased risk for all-cause and cardiovascular mortality (Diaz et al., 2005). From a physiological point of view, rHR reduction promotes a bigger and better coronary perfusion leading to a greater oxygen balance and cardiac performance. Thus, a low rHR would be of great benefit for patients with cardiovascular disease (i.e., heart failure and angina pectoris) (Hall and Palmer, 2008; Gent et al., 2015).

Beta-adrenoceptors antagonists have been used for reducing heart rate. They are indicated as first-line therapy in patients with myocardial ischemia and heart failure. However, their usage could exacerbate cardiovascular (through a negative inotropic effect) and respiratory complications, mainly in elderly patients (Rochon et al., 1999; Chaudhary et al., 2015). Consequently, a pure bradycardic agent might be useful in conditions in which beta-adrenergic blocker cannot be used due to their side effects. In this regard, ivabradine—a novel selective inhibitor of HCN channels—has emerged as a promising “pure” heart rate (HR) lowering drug (DiFrancesco and Camm, 2004; Bucchi et al., 2006).

To date, the mammalian genome encodes four HCN isoforms (HCN1 to HCN4), which relate to ion conductivity, mainly in the central nervous system and the heart (Notomi and Shigemoto, 2004; Harzheim et al., 2008). HCN4, which has been described as the main HCN channel of sinoatrial node, and the prevalent isoform expressed in the heart conduction system (Brioschi et al., 2009; Furst and D'Avanzo, 2015), is the preferential target of ivabradine (Chaudhary et al., 2015). *In vitro* studies have demonstrated that ivabradine specifically blocks the HCN pore in a low-moderate concentration range (Bucchi et al., 2002; DiFrancesco, 2010). It greatly inhibits the hyperpolarization-active current (If) and reduces the firing rate of the sinoatrial node cells at small concentrations, without influencing other ion currents (calcium and potassium) (Bois et al., 1996). Moreover, experimental and clinical studies have corroborated the use of ivabradine as a favorable therapeutic strategy, since they revealed no unwanted cardiovascular outcome (negative inotropic or lusitropic effects), preserving ventricular contractility (DiFrancesco and Camm, 2004; Sulfi and Timmis, 2006). The lack of cardiovascular side effects and the specificity of ivabradine on lowering heart rate provide relevant advantages for its clinical usage. However, little is known about the effects of ivabradine on the cardiovascular autonomic control.

HR, whose control is achieved through intrinsic and extrinsic mechanisms, respectively, by *If* pacemaker current of HCN channels and the autonomic nervous system (Verrier and Tan, 2009), are determinants of myocardial oxygen demand and may also affect myocardial perfusion. The autonomic system, which exerts reflex and tonic control over the cardiovascular homeostasis, influences the HCN voltage-dependence, changing the diastolic depolarization and, consequently, the HR (DiFrancesco, 1993). In this context, knowledge concerning the effects of chronic treatment with ivabradine on cardiovascular autonomic control has become essential. A few reports have described some autonomic effects of *If* blockers, including ivabradine, during acute endovenous treatment (Barzilai and Jacob, 2015; Dias da Silva et al., 2015). However, to our knowledge, no study has attempted to investigate the chronic effects of ivabradine on reflex autonomic control of HR and sympathetic nerve activity. Therefore, in the present study we assessed, in rats, the effects of chronic treatment with ivabradine on the cardiovascular autonomic modulation, the cardiovascular reflexes regulation (baroreflex, Bezold-Jarish reflex and chemoreflex) and tonic autonomic HR control, as well as on the RSNA.

Featuring the effects of chronic treatment with ivabradine on the HR, may provide a considerable *in vivo* understanding of its effects on the cardiovascular autonomic system. Therefore, improving our knowledge on the pharmacodynamics of this drug and providing substantial outcomes for the clinical usage of ivabradine as a cardiac medication.

## Materials and methods

### Experimental model

Experiments were performed on male Wistar rats (300 ± 10 g), supplied by the Center of Animal Science of the Federal University of Ouro Preto (UFOP). They were kept in grouped cages (*n* = 3) on a 12 h light/dark cycle, at a controlled room temperature (23°C), with free access to commercial chow and filtered water. Efforts were made to avoid any unnecessary distress to the rats, in accordance to the Brazilian Council for Animal Experimentation. All procedures were approved by the institutional ethics committee for animal research of UFOP (CEUA 2014/66; 2015/59), and were performed according to the regulations set forth by the National Institutes of Health Guidelines for the Care and Use of Laboratory Animals.

### Experimental preparation

Rats were divided in two treatment groups, respectively, vehicle and ivabradine receiving intraperitoneal (i.p.) injections of PBS (1mL/kg/day; single doses) or ivabradine (10 mg/kg/day or 2 mg/kg/day; single doses) during 7 or 8 consecutive days according to experimental design (detailed in Experimental Design Section). On the 5th day of treatment, the animals were submitted to surgical procedures, as described below.

#### Surgical procedures related to Experiment 1

Rats were anesthetized with ketamine and xylazine solution (80 mg/kg; 7 mg/kg; i.m.). Polyethylene catheters were placed into the femoral artery and vein, respectively, for cardiovascular recordings and drugs infusion, which have been described in detail elsewhere (Martins et al., 2011). Prophylactic treatment with antibiotics [Veterinary Pentabiotic—penicillin (benzatinbenzilpenicillin, procain benzilpenicillin and potassicbenzilpenicillin), streptomicyn and dihydrostreptomycin: 1 mL/kg; i.m.] and anti-inflammatory (Ketoprofen: 4 mg/kg; i.m.) drugs were performed in order to prevent postsurgical infections and inflammation, respectively (Silva et al., 2013). After 48 h of recovery from the anesthesia and surgery, rats were conducted to experimental protocol (Rodrigues-Barbosa et al., 2012).

#### Surgical procedures related to Experiment 2

Rats were anesthetized with urethane (1.2–1.4 g/kg, i.p. with supplementary doses of 0.1 g/kg i.v., if required). The adequacy of anesthesia was verified by the absence of the corneal reflex and a withdrawal response to nociceptive stimulation of a hind paw. A tracheotomy was performed to maintain and unobstructed airway, and all the animals were allowed to breathe freely. Polyethylene catheters were placed into the femoral artery and vein, respectively, for pulsatile arterial pressure recording and drug injections. The rat was mounted in a stereotaxic apparatus and the left renal nerve was exposed and prepared as described previously (Muller-Ribeiro et al., 2012; Xavier et al., 2014). Briefly, the renal nerve was exposed from a retroperitoneal approach. It was carefully separated from surrounding tissue and placed on a silver bipolar recording electrode and immersed in mineral oil for sympathetic nerve activity recording. The signals from the recording electrode were amplified and filtered (bandwidth 100–2000 Hz). The signals were then digitized (1000 samples/s) and recorded using the PowerLab system. Chart software was used to rectify and integrate the RSNA signals.

### Experimental design

As mentioned in the experimental preparation section, rats were submitted to chronic treatment during 7 and 8 days, in accordance to the previous literature (da Silva et al., 1994). All experiments started only after stabilization of physiological parameters for at least 30 min.

#### Experiment 1: the effects of chronic treatment with ivabradine (8 days) on modulatory, reflex and tonic autonomic control of heart rate (n = 14)

This experiment was conducted in non-anesthetized freely moving rats (vehicle group: PBS 1 mL/kg/day i.p.—single doses; *n* = 6 and ivabradine group: 2 mg/kg/day i.p.—single doses; *n* = 8; both during 8 days), in order to test the effects of ivabradine on the cardiovascular variability, reflex sensitivities and tonic control of HR. In this experiment, we used 2 mg/kg of ivabradine, since higher doses evoked a severe bradycardia. The adopted dose was based on previous studies (Dias da Silva et al., 2015). On the 7th day of treatment, rats were submitted to 30 min of basal period recording, which was used to quantify HR and systolic arterial pressure (SAP) variability parameters in the time—and the frequency-domain (spectral analysis), followed by the assessment of cardiovascular reflexes and cardiac autonomic tone. On the 8th day of treatment, rats were re-submitted to 30 min of basal period, followed by the measurement of cardiac autonomic tone, as will be detailed later.

In order to evaluate cardiovascular variability parameters, mean values of SAP, mean arterial pressure (MAP), diastolic arterial pressure (DAP), and pulse interval (PI) were calculated for each 20–30-min period of recording. For the cardiovascular variability study, the signals of arterial pressure (AP) were processed using a software (PRE24 software, kindly provided by Dr. Alberto Porta, University of Milan, Italy) to generate beat-to-beat time series of PI, DAP, and SAP. The variance of these values in each period was considered a variability index in the time-domain. In order to minimize the potential influence of the absolute numerical values on the calculation of variance, we have also performed the calculation of the normalized variance, which consisted in dividing each single PI, SAP, or DAP values by the mean value of the entire respective time series and multiplying the result for 100% (Sacha, 2014). Therefore, the normalized variance (calculated from the adjusted time series and expressed in %) is corrected for the mean value of the time series. The variability of PI, DAP and SAP was also evaluated in the frequency domain using an autoregressive spectral analysis method. The theoretical and analytical proceedings are described in previous studies (Malliani et al., 1991; TFESCNASPE, 1996). Briefly, beat by beat time series of PI, DAP, and SAP were divided into serial segments of 300 beats, wherein all successive segments were overlapped by 50% (150 beats) on the previous segments (Welch's method). Using stationary time series segments, autoregressive parameters were estimated using Levinson-Durbin's method, and the model order was chosen according to Akaike's criteria (Rubini et al., 1993; TFESCNASPE, 1996). Then, on each individual stationary segment of 300 beats, spectral decomposition was performed using appropriate autoregressive software. The normalization procedure, applied only to the variability of the PI, was performed by dividing the power of the low frequency component (low frequency—LF, 0.20–0.750 Hz) or high frequency (high frequency—HF, 0.75–3.00 Hz) by total spectral power, which is subtracted from the power of the very low frequency band (very low frequency—VLF, 0.01 to 0.20 Hz), and multiplying the result by 100 (Rubini et al., 1993). The spectral parameters obtained for each individual stationary segment of 300 beats were averaged, and the average values resulting from 30 min of recording were calculated for each animal.

In order to assess the baroreflex sensitivity, we administered intravenous bolus injection (i.v.) of phenylephrine (2, 4, and 8 μg/kg) and sodium nitroprusside (4, 8, and 16 μg/kg, i.v.) for calculating the ΔHR/ΔMAP index (Oliveira et al., 2005), which was obtained through the mean of the three doses. We also administered phenylbiguanide (1.25; 2.5 and 5 μg/kg, i.v.) and potassium cyanide (80 and 160 μg/kg, i.v.) to evaluate, respectively, the Bezold-Jarish reflex and chemoreflex sensitivities (Penitente et al., 2007; Bezerra et al., 2011). All aforementioned i.v. injections were performed every 5 min. Subsequently, to evaluate the ivabradine influence on the tonic autonomic control of the heart, we also performed the sympathetic and vagal autonomic blockade after propranolol (5 mg/kg, i.v.) and methylatropine (4 mg/kg, i.v.) injections, respectively, to calculate the sympathetic and vagal effects, as well as the intrinsic HR and tonic sympathovagal index (Goldberger, 1999). The autonomic blockers were administered in a random sequence with a 15 min interval between them, on the 7th and 8th days of treatment. After double blockade, the cardiovascular recordings lasted for 15 min. Briefly, the sympathetic effect was considered as the difference between the HR after sympathetic blockade and resting HR (Sympathetic effect = HR after sympathetic blockade - resting HR). Vagal effect was calculated as the difference between HR after vagal blockade and resting HR (Vagal effect = HR after vagal blockade - resting HR). The tonic sympathovagal index was obtained as the ratio between resting and intrinsic HR, considering that the intrinsic HR (iHR) was the HR obtained after double autonomic blockade (Goldberger, 1999).

#### Experiment 2: the effects of chronic treatment with ivabradine (7 days) on heart rate (HR), mean arterial pressure (MAP) and renal sympathetic nerve activity (RSNA) (n = 10)

This experiment was conducted in urethane-anesthetized rats (vehicle group: PBS 1 mL/kg/day i.p.; single doses; *n* = 5 and ivabradine group: 10 mg/kg/day i.p.; single doses; *n* = 5, both during 7 days), in order to test the effects of ivabradine on HR, MAP, and RSNA. The ivabradine dose was chosen based on earlier studies (Du et al., 2004; Luszczki et al., 2013). For this purpose, such parameters were recorded during 60 min. The RSNA signal was amplified (10 K), filtered (100–1000 Hz), displayed on an oscilloscope and monitored via an audio-amplifier. The filtered nerve activity signal was rectified, integrated (resetting every second), displayed online and acquired using Powerlab 4/20 LabChart 7.1 (ADInstruments, Sydney, Australia). All data were digitized at 1 kHz. The noise of the recording system was determined post mortem (urethane: 0.5 mL; i.v) and subtracted from the RSNA values obtained during the experiment (Muller-Ribeiro et al., 2012; Xavier et al., 2014). The quantitation of spikes/second was conducted using a previously described methodology (Gomes da Silva et al., 2012). Body temperature was monitored since the beginning of surgical procedure using a rectal thermometer and maintained in the range of 37–37.5°C using a heating pad (Xavier et al., 2014).

### Statistical analysis

Regarding Experiment 1, baseline values of HR and SAP variability parameters were compared. In addition, for reflexes and autonomic tone studies, baseline values of HR and MAP were obtained by averaging the 1 min-period that preceded drugs injections. Maximum changes (mean ± SEM) were calculated using the peak response after drugs injections (for cardiovascular reflexes analysis) or using the last 1 min-period corresponding to each autonomic blockade recording (for autonomic tone analysis). The effects between groups (ivabradine 2 mg/kg vs. vehicle) were compared using Student's unpaired *t*-test.

In relation to the Experiment 2, data were obtained by averaging the values of the whole recording. They were expressed as absolute values and reported as mean ± SEM. The effects between groups (ivabradine 10 mg/kg vs. vehicle) were compared using Student's unpaired *t*-test.

Prism 5.0 (GraphPad Software, La Jolla, CA, USA) was used to analyze all data. The significance level was set at *p* < 0.05.

## Results

### Experiment 1: chronic treatment with ivabradine (8 days) did not change cardiovascular variability as well as the reflex and tonic autonomic control of heart rate in non-anesthetized rats

**Figure 1:**
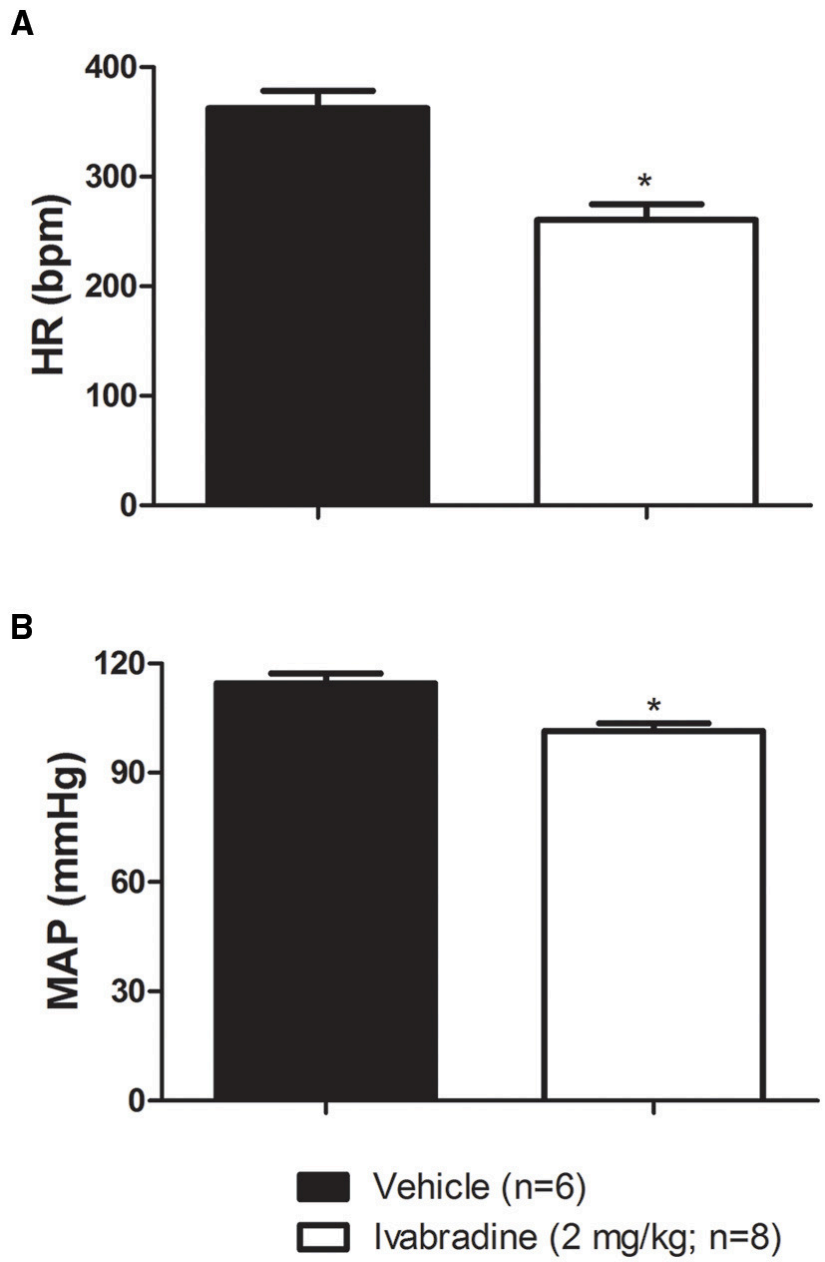
**Values of (A) resting heart rate (HR, bpm) and (B) mean arterial pressure (MAP, mmHg) in non-anesthetized Wistar rats submitted to vehicle (PBS 1 mL/kg/day i.p.; single dose; *n* = 6) or ivabradine (2 mg/kg/day i.p.; single dose; *n* = 8) treatment during 8 days**. Bars represent mean ± SEM. ^*^p < 0.05, vehicle (VEH-black) vs. ivabradine (IVA- White); Student's unpaired *t*-test.

As expected, compared with vehicle-treated group (VEH), the ivabradine-treated rats (IVA: 2 mg/kg/day; i.p.) presented lower resting (VEH: 362 ± 16 bpm vs. IVA: 260 ± 14 bpm, *p* = 0.0005; Figure 1A) and intrinsic HR (VEH: 369 ± 9 bpm vs. IVA: 326 ± 11 bpm, *p* = 0.0146; Figure 3D). Ivabradine treatment also decreased resting MAP (VEH: 115 ± 3 mmHg vs. IVA: 102 ± 2 mmHg, *p* = 0.0020; Figure 1B).

Heart rate variability analysis by means of spectral autoregressive decomposition of pulse interval (PI) time-series in all experimental groups is summarized in Table 1. Treatment with ivabradine significantly increased variance and absolute values of LF and HF spectral components, without any changes in normalized LF and HF parameters and LF/HF ratio in ivabradine-treated rats when compared to vehicle-treated animals (Table 1). Systolic arterial pressure variability analysis is summarized in Table 2. Despite of the significantly lower values of SAP, no changes in SAP variability parameters were observed in ivabradine-treated rats when compared to vehicle-treated animals (Table 2). The behavior of diastolic arterial pressure variability was similar to the SAP variability (data not shown).

**Table 1:** **Mean values (±SEM) of pulse interval (PI), variance and VLF, LF, and HF spectral components of pulse interval variability in non-anesthetized Wistar rats from vehicle or ivabradine-treated rats**.

	**Vehicle (*n* = 6)**	**Ivabradine (*n* = 8)**
PI (ms)	166.0 ± 6.4	232.1 ± 7.9^*^
Variance (ms^2^)	22.3 ± 6.7	70.1 ± 10.5^*^
Normalized Variance (%)	6.7 ± 1.2	18.9 ± 3.2^*^
VLF (ms^2^)	10.6 ± 3.6	22.2 ± 4.8
LF (ms^2^)	3.2 ± 3.1	15.2 ± 4.1^*^
LF (nu)	24.2 ± 4.6	29.8 ± 6.4
HF (ms^2^)	8.4 ± 4.7	32.4 ± 7.5^*^
HF (nu)	75.1 ± 3.7	69.2 ± 5.8
LF/HF	0.31 ± 0.09	0.35 ± 0.13

*PI, Pulse interval; VLF, very low frequency component; LF, low frequency component; LF (nu), LF in normalized units; HF, high frequency component; HF (nu), HF in normalized units*.

* *p < 0.05 vs. vehicle*.

**Table 2:** **Mean values (±SEM) of systolic arterial pressure (SAP), variance and VLF, LF and HF spectral components of arterial pressure variability in non-anesthetized Wistar rats from vehicle or ivabradine-treated rats**.

	**Vehicle (*n* = 6)**	**Ivabradine (*n* = 8)**
SAP (mmHg)	142.2 ± 3.4	128.5 ± 3.1^*^
Variance (mmHg)^2^	15.3 ± 4.1	17.6 ± 6.2
Normalized Variance (%)	11.2 ± 1.4	15.1 ± 2.3
VLF (mmHg)^2^	5.4 ± 0.7	6.1 ± 1.6
LF (mmHg)^2^	6.2 ± 1.5	6.8 ± 2.0
HF (mmHg)^2^	3.3 ± 2.0	4.1 ± 1.8
LF/HF	1.9 ± 0.8	1.6 ± 1.8

*SAP, Systolic arterial pressure; VLF, very low frequency component; LF, low frequency component; HF, high frequency component*.

* *p < 0.05 vs. vehicle*.

**Figure 2:**
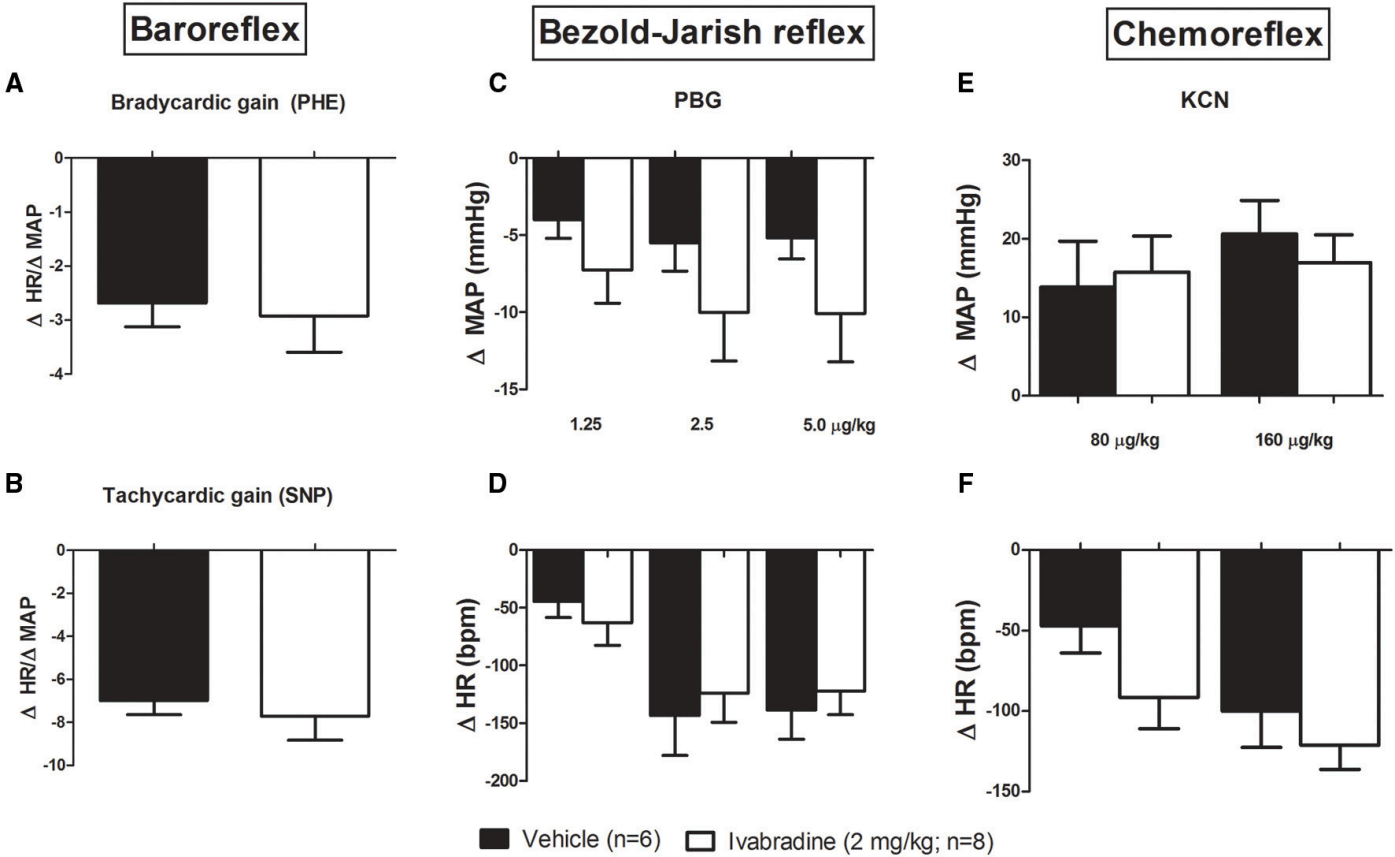
**Effects of chronic treatment with ivabradine on the reflex control of the heart rate in non-anesthetized Wistar rats. (A)** Baroreflex bradycardic gain and **(B)** baroreflex tachycardic gain, respectively, induced by phenylephrine (PHE: 2, 4, and 8 μg/kg) and sodium nitroprusside (SNP: 4, 8, and 16 μg/kg, i.v.) injections. Baroreflex gains were obtained by ΔHR/ΔMAP index (bpm/mmHg), which was calculated through the mean of three used doses. Changes in mean arterial pressure (MAP, mmHg) and heart rate (HR, bpm) induced by **(C,D)** phenylbiguanide (PBG: 1.25; 2.5, and 5 μg/kg, i.v.) and **(E,F)** potassium cyanide (KCN: 80 and 160 μg/kg, i.v.) injections were performed to assess the Bezold-Jarish reflex and chemoreflex regulation, respectively, in non-anesthetized Wistar rats submitted to vehicle (PBS 1 mL/kg/day i.p.; single dose; *n* = 6) or ivabradine (2 mg/kg/day i.p.; single dose; *n* = 8) treatment during 8 days. Bars represent mean ± SEM of vehicle (VEH—black) and ivabradine (IVA—white) groups. *p* > 0.05, VEH vs. IVA (Student's unpaired *t*-test).

Regarding the possible influence of the chronic treatment with ivabradine on HR baroreflex control, we administered intravenous injection (i.v.) of phenylephrine (PHE: 2, 4, and 8 μg/kg) and sodium nitroprusside (SNP: 4, 8, and 16 μg/kg, i.v.) to assess the baroreflex sensitivity. The baroreflex bradycardic and tachycardic gains were calculated through ΔHR/ΔMAP index. No changes were observed in bradycardic (VEH:−2.7 ± 0.4 bpm/mmHg vs. IVA:−2.9 ± 0.8 bpm/mmHg, *p* = 0.8025; Figure 2A) and tachycardic gains (VEH:−6.9 ± 0.6 bpm/mmHg vs. IVA:−7.7±1.1 bpm/mmHg, *p* = 0.6432; Figure 2B) between groups. We also administered phenylbiguanide (PBG: 1.25; 2.5 and 5 μg/kg, i.v.) and potassium cyanide (KCN: 80 and 160 μg/kg, i.v.) to assess the Bezold-Jarish reflex and chemoreflex regulation, respectively. The treatment with ivabradine during 8 days did not modify the cardiovascular responses to Bezold-Jarish reflex activation, since all PBG injected doses induced similar hypotension and bradycardia in VEH and IVA groups (**ΔMAP: PBG 1.25**−VEH:−4 ± 1 mmHg vs. IVA:−7 ± 2 mmHg, *p* = 0.2525; **PBG 2.5**−VEH:−5.5 ± 2 mmHg vs. IVA:−10 ± 3 mmHg, *p* = 0.2303; **PBG 5**−VEH:−5 ± 1 mmHg vs. IVA:−10 ± 3 mmHg, *p* = 0.1318; Figure 2C); (**ΔHR: PBG 1.25**−VEH:−45 ± 14 bpm vs. IVA: −63 ± 20 bpm, *p* = 0.5; **PBG 2.5**−VEH:−143 ± 35 bpm vs. IVA:−124 ± 25 bpm, *p* = 0.2848; **PBG 5**−VEH:−139 ± 25 bpm vs. IVA:−122 ± 20 bpm, *p* = 0.3199; Figure 2D). Similarly, the ivabradine treatment did not change the chemoreflex responsiveness. The amplitude of hypertensive and bradycardic responses evoked by KCN injections were similar in both groups (**ΔMAP: KCN 80**−VEH: 14 ± 6 mmHg vs. IVA: 16 ± 5 mmHg, *p* = 0.4259; **KCN 160**−VEH: 21 ± 4 mmHg vs. IVA: 17 ± 4 mmHg, *p* = 0.5162; Figure 2E); (**ΔHR: KCN 80**−VEH:−66 ± 23 bpm vs. IVA:−92 ± 19 bpm, *p* = 0.2018; **KCN 160**−VEH:−87 ± 22 bpm vs. IVA:−121±15 bpm, *p* = 0.1087; Figure 2F).

**Figure 3:**
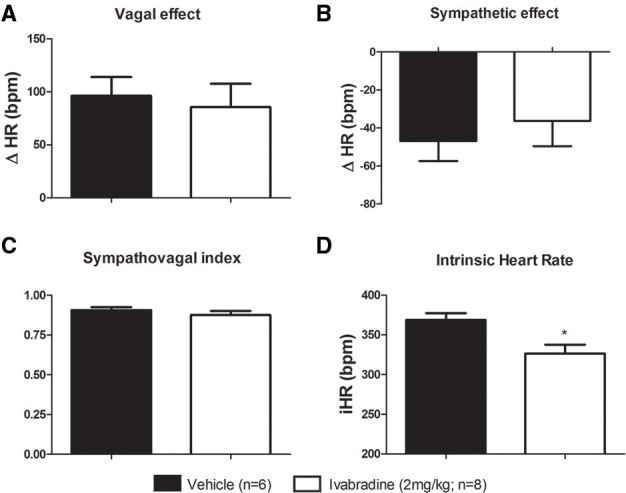
**Effects of chronic treatment with ivabradine on the tonic autonomic control of the heart rate in non-anesthetized Wistar rats. (A)** Vagal and **(B)** sympathetic effects were obtained, respectively, by the difference between vagal blockade (by methylatropine: 4 mg/kg, i.v.) or sympathetic blockade (by propranolol: 5 mg/kg, i.v.) and resting HR. **(C)** Sympathovagal balance was expressed by the tonic sympathovagal index, which is the ratio between resting and intrinsic HR. **(D)** Intrinsic HR (iHR, bpm) —the HR was obtained after autonomic double blockade. All aforementioned parameters were evaluated in non-anesthetized Wistar rats submitted to vehicle (PBS 1 mL/kg/day i.p.; single dose; *n* = 6) or ivabradine (2 mg/kg/day i.p.; single dose; *n* = 8) treatment during 8 days. Bars represent mean ± SEM of vehicle (VEH—black) and ivabradine (IVA—white) groups. ^*^p < 0.05, VEH vs. IVA (Student's unpaired *t*-test).

In order to evaluate the influence of ivabradine chronic treatment on the tonic autonomic control of the heart, we performed the vagal and sympathetic autonomic blockade through methylatropine (4 mg/kg, i.v.) and propranolol (5 mg/kg, i.v.) injections, respectively, to calculate the vagal and sympathetic effects, as well as the tonic sympathovagal index. No differences on vagal (**ΔHR;** VEH: 96 ± 18 bpm vs. IVA: 86 ± 22 bpm, *p* = 0.3571; Figure 3A) neither on sympathetic effects (**ΔHR;** VEH: −47 ± 11 bpm vs. IVA: −36 ± 13 bpm, *p* = 0.2724; Figure 3B) were observed between VEH and IVA groups. Additionally, the ivabradine treatment did not alter the sympathovagal balance, expressed by tonic sympathovagal index (VEH: 0.91 ± 0.02 vs. IVA: 0.88 ± 0.03, *p* = 0.3494; Figure 3C).

#### Experiment 2: chronic treatment with ivabradine (7 days) reduced heart rate (HR), without significant effects on mean arterial pressure (MAP) and renal sympathetic nerve activity (RSNA) in urethane-anesthetized rats

**Figure 4:**
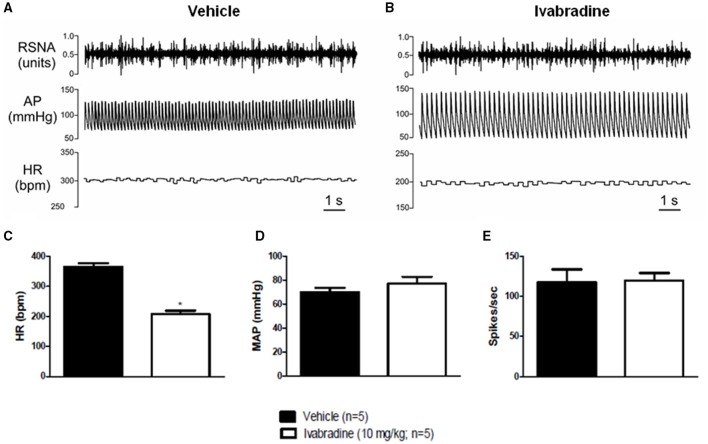
**Effects of chronic treatment with ivabradine on heart rate, mean arterial pressure and renal sympathetic nerve activity in urethane-anesthetized Wistar rats**. **(A)** Representative records of vehicle (PBS 1 mL/kg/day i.p.; single dose; *n* = 5) or **(B)** ivabradine-induced changes (10 mg/kg/day i.p.; single dose; *n* = 5) in heart rate (HR, bpm), mean arterial pressure (MAP, mmHg) and renal sympathetic nerve activity (RSNA, spikes/sec) after 7 days of treatment. Bars represent mean ± SEM of resting HR **(C)**, MAP **(D)**, and RSNA **(E)** in vehicle (VEH—black) and ivabradine (IVA—white) groups. ^*^p < 0.05, VEH vs. IVA (Student's unpaired *t*-test).

Representative records **(A,B)** and changes in HR **(C)**, MAP **(D)** and RSNA **(E)** induced by the chronic treatment with ivabradine were shown in Figure 4. Compared with vehicle treatment, the ivabradine administration (10 mg/kg/day; i.p.) during 7 consecutive days markedly reduced resting HR (VEH: 364 ± 12 bpm vs. IVA: 207 ± 11 bpm, *p* < 0.0001; Figure 4C). However, such ivabradine treatment did not change MAP (VEH: 70 ± 4 vs. IVA: 77 ± 6 mmHg, *p* = 0.3293; Figure 4D) and RSNA (VEH: 117 ± 16 vs. IVA: 120 ± 9 spikes/sec, *p* = 0.9100; Figure 4E).

## Discussion

In this study, we evaluated the effects of chronic treatment with ivabradine—a “pure” HR lowering drug which selectively inhibits the pacemaker HCN channels—on the autonomic control of the HR in rats. Our results showed that, in healthy animals, a long-term ivabradine administration reduced resting HR without promoting any change on the cardiovascular reflexes and tonic autonomic regulation of the heart, as well as on RSNA. As no study has investigated this set of variables to date, our data provides new *in vivo* insights about the chronic effects of ivabradine on the cardiovascular autonomic system.

Exploring whether chronic treatment with ivabradine could influence the autonomic regulation of the HR, we assessed its effects on the reflex and on the tonic autonomic control. In the present investigation, performed in freely moving rats, ivabradine treatment (2 mg/kg/day) during 8 days induced a significant reduction in resting and intrinsic HR. Furthermore, it also significantly decreased resting MAP, which could be ascribed to a cardiac output fall due to a marked ivabradine-induced bradycardia. It is plausible to infer that, since we did not observe any ivabradine-induced RSNA and autonomic changes, which indirectly suggest no effect on the peripheral resistance. The ivabradine-induced bradycardia (28%) was greater than that achieved in other experimental (Dias da Silva et al., 2015) and clinical studies (15–20%) (Fox et al., 2008; Swedberg et al., 2010). The discrepancy between the bradycardia magnitudes may be explained, at least, by two reasons. Firstly, the mentioned experimental report was performed on anesthetized rats, which were submitted to acute ivabradine injection (Dias da Silva et al., 2015). Secondly, there is a plausible difference between the pharmacodynamic of ivabradine in rats and humans, which exhibit lower HR values. The ivabradine blockade is current-dependent and favored by depolarization, when the drug molecules are impelled to their intracellular binding site by the inward flow of permeating cations (sodium and potassium). In addition, ivabradine binds to HCN channels in open state, whose configuration depends on the attachment of the cyclic adenosine monophosphate (cAMP). Consequently, ivabradine presents upper activity when cAMP levels are high, such as in adrenergic stimulation that results in higher HR. It is a particular property, which provides its strong use-dependent action and suggests that its rate-reducing efficiency might be enhanced at high rates (Bucchi et al., 2002; DiFrancesco, 2005), as observed in non-anesthetized rats. Although, ivabradine-induced bradycardia observed in our study was not comparable to those considered appropriated to therapeutic action, it was crucial for our objective to obtain a greater bradycardia, since it assured a worst scenario for its potential influence on autonomic system.

We also analyzed the effects of chronic treatment with ivabradine on cardiovascular reflex control (baroreflex, Bezold-Jarish reflex and chemoreflex). The ivabradine treatment did not affect the bradycardic and tachycardic gains of baroreflex, neither the hypotensive and bradycardic responses evoked by Bezold-Jarish reflex activation, as well as the hypertensive and bradycardic responses induced by peripheric chemoreceptors stimulation. To our knowledge, it was the first time that these reflexes were studied in non-anesthetized rats submitted to long-term use of ivabradine. A few reports leading with this subject investigated the acute intravenous injection effects of HCN blockers in rats (Kruger et al., 2000; Dias da Silva et al., 2015). One study, in which acute zatebradine i.v. injection was conducted in non-anesthetized sham-rats, revealed an increase in arterial baroreflex sensitivity (Kruger et al., 2000). However, a recent study from our group performed in anesthetized rats, that received acute ivabradine i.v. injection, showed that ivabradine did not alter the baroreflex sensitivity (Dias da Silva et al., 2015). As mentioned above, chronic treatment, during 7–8 days, could permit a resetting of cardiovascular autonomic reflexes to a new hemodynamic context.

In order to deepen the understanding about the influence of chronic use of ivabradine on autonomic regulation of the HR, we also evaluated its effects on modulatory and tonic autonomic control. For that, we performed HR variability analysis, and vagal and sympathetic autonomic blockade, respectively. We also evaluated the tonic sympathovagal index, which is a validated methodology to assess the vagal and sympathetic cardiac tonic balance (Goldberger, 1999). Regarding to HR variability analysis, we observed in ivabradine-treated animals a markedly higher value of variance, a time–domain index, which was accompanied to higher values in LF and HF spectral components, without any changes in normalized LF and HF components, as well as in LF/HF ratio. Even though, at the first glance, these findings could suggest an increased sympathetic (higher LF component) and parasympathetic modulation (higher variance and HF component). The normalization procedure and LF/HF ratio show a balanced autonomic modulation. As previously reported by our group (Dias da Silva et al., 2015) and others (Rocchetti et al., 2000; Zaza and Lombardi, 2001; Monfredi et al., 2014), the increase in all spectral components (expressed in absolute units) and in the variance (sum of the individual spectral components) can be considered, since these parameters are intrinsically dependent on mean interbeat interval (pulse interval), which was directly increased by ivabradine more than possible changes in the autonomic influences toward sinus node. In fact, our results of autonomic blockade with atropine and propranolol seem to reinforce this idea, since no differences were observed on vagal and sympathetic effects. Additionally, the tonic sympathovagal index was not affected by ivabradine treatment. It was < 1 in vehicle and ivabradine groups, indicating a vagal dominance in both (Goldberger, 1999). Taken together, these data suggest that, in rats, the sustained use of ivabradine did not alter the autonomic balance to the heart. Despite of that, as showed by Mangin et al. (1998), the intrinsic dependency of variance and spectral parameters on mean HR does not rule out the possibility that these parameters may be also modulated by neural influences, which probably could be the case under several conditions of real autonomic imbalance, as observed in many cardiovascular disease conditions (Malliani et al., 1991; TFESCNASPE, 1996; Schwartz and De Ferrari, 2011). Despite of a small decrease in arterial pressure, SAP variability data did not show any difference between both vehicle and ivabradine groups. LF component of SAP variability has been considered an indirect marker of vascular sympathetic modulation, suggesting that chronic treatment with ivabradine does not change sympathetic activity not only to the heart but also to the peripheral vessels. However, given the large standard errors of means observed on SAP variability parameters, the lack of differences between vehicle and ivabradine treatment should be interpreted cautiously and not as a definitive statement that ivabradine does not change arterial pressure variability. More experiments with a higher number of cases in a near future should be performed to clarify this issue.

In order to further understanding ivabradine actions on sympathetic control, we assessed the sympathetic nervous system responsiveness to long-term treatment with ivabradine by a direct methodology. Thus, we analyzed the effects of chronic injections of ivabradine (10 mg/kg/day; during 7 days) on RSNA, as well as in HR and MAP in urethane-anesthetized rats. Recordings of RSNA are technically more amenable in anesthetized than in freely moving animals (Xavier et al., 2014), however the cardiovascular autonomic control is affected by anesthetics. Urethane presents minor interference with autonomic activity, but reduces the sympathetic tone whereas it seems to leave the parasympathetic tone relatively intact (Shimokawa et al., 1998; Bencze et al., 2013). Taking this into account, as Experiment 2 was performed under urethane-anesthesia, we used a ivabradine dose 5-fold higher to that administered in Experiment 1 because it was substantial to assure a enough severe scenario to evaluate the potential influence of ivabradine on autonomic control. Ivabradine treatment reduced resting HR, without affecting MAP, corroborating previous studies (Du et al., 2004; Verrier et al., 2014; Gent et al., 2015). Furthermore, it did not change RSNA. RSNA is closely correlated with arterial pressure and is believed to be a reliable indicator of overall sympathetic vasomotor activity (Burgess et al., 1997). This finding is consistent with the SAP and HR variabilities parameters observed in non-anesthetized rats, strongly suggesting that long-term ivabradine administration does not compromise the sympathetic autonomic regulation. A recent study by our group showed that, in rats, ivabradine-induced bradycardia was associated with increased cardiac sympathetic nerve activity, resulting from baroreceptor unloading (Dias da Silva et al., 2015). Additionally, another study reported that, in sham-rats, zatebradine (another HCN blocker) increases the heart rate variability—a marker of autonomic modulation of HR (Kruger et al., 2000). However, such data methodologically differs from ours in some aspects: (i) they refer to acute i.v. injections while in our study the ivabradine effects were assessed 7–8 days after continuous treatment, a time frame long enough to induce baroreceptor resetting and, (ii) the second report used a HCN blocker less specific than ivabradine. Ivabradine, which is an open channel-required blocker, reaches its binding site by entering in the HCN pore from an intracellular side, being able to specifically block the pore channel in a low-moderate concentration range (Bucchi et al., 2002).

Corroborating the widespread idea that ivabradine is a promissor cardiac medication, three current clinical studies have shown that acute treatment with ivabradine did not alter the HR and blood pressure variabilities and baroreflex sensitivity, suggesting no implications on the sympatho-vagal balance in healthy men (Heusser et al., 2016; Schroeder et al., 2016) and in postural tachycardia syndrome patients (Barzilai and Jacob, 2015). In addition, some studies have reported that ivabradine exerts no side effects on the: (i) conductivity and refractoriness (in atrium, atrioventricular node, His–Purkinje system and ventricles), (ii) left ventricular ejection fraction, (iii) stroke volume and (iv) some ECG measurements (corrected QT, PR, and QRS intervals) (DiFrancesco and Camm, 2004), reinforcing the advantage and viability of ivabradine use to the detriment of others reducing HR drugs, such as beta-blockers. Blockade of beta receptors, which are found throughout the heart, can promote a beneficial reduction of HR, though it could trigger side effects due to its presence in several organs, including in the cardiovascular and respiratory systems (Tattersfield, 1991; Bois et al., 1996; Sulfi and Timmis, 2006). Beta-blockers also slow the *If* current via the reduction in sympathetic activity and cAMP formation, while ivabradine acts specifically inhibiting *If* current (Sulfi and Timmis, 2006).

In accordance to all aforementioned, our data indicates that long-term treatment with ivabradine, in healthy rats, reduces the resting and intrisic heart rate, without compromising: (i) baroreflex sensitivity, (ii) Bezold-Jarish reflex control, (iii) chemoreflex resposiveness, (iv) BP and HR variabilities, (v) vagal and sympathetic tones, (vi) tonic sympathovagal index and (vii) RSNA. Characterizing the actions of chronic treatment with ivabradine on the cardiovascular autonomic control in rats can provide an additional understanding of its effects on cardiovascular autonomic system, and then significantly improve our knowledge to support the ivabradine clinical use.

## Author contributions

FS drafted the work and substantially contributed to work design, as well as, acquired, analyzed and interpreted the all data. FP acquired the data and also substantially contributed to work design and to analysis and interpretation of the all data. FM substantially contributed to acquisition and analysis of the RSNA data, HC substantially contributed to Bezold Jarish reflex data acquisition. MF, RD, KC, GF, ET, and MS substantially contributed to all data interpretation, NM, VD, and DC designed the work, and substantially contributed to analysis and interpretation of the all data. All authors revised the work critically, approved the final version to be published and declared accountable for all aspects of the work.

## Funding

This study was supported by the Conselho Nacional de Desenvolvimento Científico e Tecnológico (CNPq, grant #400851/2014-8 for VD), Coordenação de Aperfeiçoamento de Pessoal de Nível Superior (CAPES), Fundação de Amparo à Pesquisa de Minas Gerais (FAPEMIG), Universidade Federal de Ouro Preto (UFOP) and Universidade Federal do Triângulo Mineiro (UFTM), Brazil.

### Conflict of interest statement

The authors declare that the research was conducted in the absence of any commercial or financial relationships that could be construed as a potential conflict of interest. The reviewer MF and handling Editor declared their shared affiliation, and the handling Editor states that the process nevertheless met the standards of a fair and objective review. The reviewer DC declared a shared affiliation, though no other collaboration, with one of the author KC to the handling Editor, who ensured that the process nevertheless met the standards of a fair and objective review.
